# Increased *MYD88* blood transcript in a mouse model of Alzheimer’s disease

**DOI:** 10.1186/s12868-022-00699-8

**Published:** 2022-03-11

**Authors:** Catalina Anca Cucos, Maria Dobre, Elena Mihaela Dragnea, Gina Manda, Elena Milanesi

**Affiliations:** 1 Laboratory of Radiobiology, “Victor Babes” National Institute of Pathology, Bucharest, Romania; 2 Laboratory of Histopathology and Immunohistochemistry, “Victor Babes” National Institute of Pathology, Bucharest, Romania

**Keywords:** MYD88, Mouse model, Alzheimer’s disease, Rivastigmine

## Abstract

**Background:**

Neuroinflammation plays a prominent role in Alzheimer’s disease (AD), both in pathogenesis and disease progression. It has been shown that TLR/MYD88 signaling is involved in the chronic low-grade sterile inflammation associated with AD. Several studies have evidenced high levels of MYD88 in the brain of patients and animal models of AD, but no study has assessed so far its levels in blood.

**Methods:**

In this study we evaluated the blood mRNA levels of *MYD88* in a mouse model of AD, and also the putative effect of Rivastigmine treatment on *MYD88* expression. Twenty-eight transgenic APP/TAU mice (AT) and twenty-two control C57/BL6j mice (WT) were included in this study, out of which five transgenic AT and five WT mice were treated with Rivastigmine.

**Results:**

Increased *MYD88* transcript in the whole blood from AT mice as compared to WT controls was found, which seems to increase in time due to disease progression and not to aging. This finding suggests that blood leukocytes are primed to develop TLR/MYD-mediated inflammatory processes. Moreover, results indicate that *MYD88* blood levels were not modulated by the diseases-specific treatment with Rivastigmine.

**Conclusions:**

Our results suggest that *MYD88* might be a promising blood biomarker to monitor AD progression.

**Supplementary Information:**

The online version contains supplementary material available at 10.1186/s12868-022-00699-8.

## Background

Sterile inflammation of the central nervous system (CNS), was shown to underlie various neurodegenerative disorders including amyotrophic lateral sclerosis (ALS), Parkinson disease (PD) and Alzheimer’s disease (AD) [1]. Among these diseases, AD has been indicated as the condition where neuroinflammation has the most prominent role, being involved both in pathogenesis and progression [2]. Microglia and astrocytes are the main local immune cells involved in this process by producing pro-inflammatory factors like cytokines and chemokines, along with reactive oxygen species. Brain-infiltrating blood leukocytes and capillary endothelial cells also contribute to neuroinflammation [2]. Depending on the duration and intensity of neuroinflammation, both positive and negative outcomes have been described. Thus, transiently low and medium inflammation leads to enhanced plasticity, tissue repair and neuroprotection, while chronic low- and high-level inflammation seems to be associated with reduced plasticity, neuronal damage and cognitive impairment in neurodegenerative diseases and aging [3].

One of the contributing inflammation mechanisms is related to Toll-like receptors (TLRs) signaling [4]. TLRs are transmembrane proteins expressed by both immune and non-immune cells. Intracellular TLRs mainly recognize nucleic acids derived from bacteria and viruses, while cell surface TLRs interact with microbial membrane components as well as with damage-associated molecular patterns (DAMPs) [5]. In the AD brain, besides pathogens, abnormally folded proteins and protein aggregates, such as TAU and amyloid beta, have been shown to activate TLRs [6], and to trigger local inflammatory responses that affect synaptic plasticity, microglial activity and TAU phosphorylation [7]. In this context, DAMPs have been shown to be associated with neuronal dysfunction, leading to neuronal death in AD. For instance, signaling events related to DAMPs-induced unfolded protein response may regulate the expression of different AD-related proteins and early processes of β-amyloid precursor protein (APP) maturation. Hence, some DAMPs, including neuronal stress-induced HSP72 and TLR2, 4 and 9, have been reported to be involved in AD-associated neuroinflammation [8].

Except for TLR3, the myeloid differentiation factor-88 adaptor protein (MYD88) mediates the intra-cellular signaling of all other TLRs [9]. MYD88 binds to the cytoplasmic region of TLRs and activates the interleukin-1 receptor-associated kinase (IRAK) family, leading to a variety of functional outputs, including the activation of the nuclear factor-kappa B (NFκB) [10], a key mediator of inflammatory responses that regulates the survival, activation and differentiation of innate immune cells and the inflammatory Th1 and Th17 cells [11]. Moreover, it has been demonstrated that TLR/MYD88 signaling plays an important role in sustaining the chronic low-grade sterile inflammation associated with AD [12].

Post-mortem brain studies in AD patients and animal models have evidenced high levels of MYD88 in the brain, and it has been shown that experimental MYD88 deficiency ameliorates both β-amyloidosis and cognitive functions [13–15]. The research on MYD88 has remained confined to the AD brain, and no study has assessed so far its levels in blood leukocytes. Therefore, the aim of the present study was to evaluate the blood mRNA levels of *MYD88* in a mouse model of AD, and to evaluate the putative effect of AD-specific medication on gene expression.

## Methods

### Transgenic mouse model of AD

Double transgenic APP/TAU (AT) mice were used as animal model of AD. These mice express the human genes APP and TAU under the control of the mouse *Thy1* gene promoter. APP/TAU mice were obtained by crossing for more than eight generations mice presenting the human APP-V717I mutation with TAUP301L transgenic mice, both having a C57/BL6j background [16]. The characteristics of the APPV717I and TAUP301L transgenic mice have been previously described [17]. The double transgenic APP/TAU (AT) mouse model shows a combined amyloid and TAU-pathology, which mimics the pathology of AD patients, including diffuse and senile plaques, vascular amyloid and neurofibrillary tangles in brain [18]. Amyloid accumulation begins at an early age, intracellularly, decreases with age and is progressively replaced by extracellular amyloid deposits, first in the form of diffuse plaques (10–12 months), followed by senile plaques (12–15 months) and deposits of amyloid at the vascular level (15–18 months). The formation of Tau protein fibrils, especially in the anterior brain and hippocampus, occurs around the age of 13 months [18]. Twenty-eight AT mice and twenty-two control WT mice, all on C57/BL6j background, with a mean age of 55.26 ± 6.44 weeks, were investigated. The transgenic AT and WT mice were homogeneous for age and sex (Table 1). Mice were group-housed in simple cages under standard conditions (normal 12 h light/dark cycle, constant temperature and humidity), with *ad libitum* access to food and water. The study was conducted according to the guidelines of the European Directive 2010/63/EU and approved by the Ethics Committee of “Victor Babes” National Institute of Pathology, Bucharest, Romania, authorization No. 39/11.04.2017, and by the Romanian National Authority for Veterinary Research, authorization No. 385/09.02.2018.

**Table 1:** Age and sex of the experimental animals, transgenic AT and control WT mice

	AT (N = 28)	WT (N = 22)	Significance
Age (weeks)	55.42 ± 6.59	55.04 ± 6.39	p = 0.837
Sex	10 F/18 M	9 F/13 M	χ^2^ = 0.141; p = 0.707

Age was expressed as mean value ± SD. Comparison between transgenic mice (AT) and controls (WT) were evaluated using the t-test for continuous variables and the χ^2^ test for categorical variables

### Rivastigmine treatment

Five transgenic AT mice and five control WT mice were treated with Rivastigmine (Sandoz, 2 mg/mL oral solution) by daily oral gavage (0.75 mg/Kg) for 50 days, with intermediary blood collection at 20 days of treatment.

### Blood collection

Blood was collected by retro-orbital sinus sampling in PAXgene RNA stabilizer solution (Qiagen). The volume of the collected blood was between 140 and 180 µl, according to the body weight of the mice. Blood was collected before (T0), during (T1, 20 days) and at the end (T2, 50 days) of Rivastigmine treatment.

### Gene expression analysis

RNA isolation was performed using the modified PAXgene method [19], and was quantified using a NanoDrop 2000 spectrophotometer (Thermo Scientific). Reverse transcription of 500 ng total RNA was performed using the High-Capacity cDNA Reverse Transcription Kit (Thermo Scientific) according to the manufacturer’s protocol. The expression level of *MYD88* was assessed by qPCR on 7500 Fast Real-Time PCR System (Applied Biosystems), using the primers described in Table 2. *MYD88* levels were normalized on the geometric mean of two reference genes, GAPDH and TBP (Table 2). The stability of the reference genes was assessed with the RefFinder algorithm (http://leonxie.esy.es/RefFinder/) [20]. The gene expression levels of *MYD88* were presented as 2^−∆CT^ values and are available in the Additional file 1: 10.7910/DVN/XRQWSE.

**Table 2:** Primer sequences used in this study: *MYD88* Myeloid differentiation factor-88, *GAPDH* Glyceraldehyde 3-phosphate dehydrogenase, *TBP* TATA-binding protein

Gene	Reverse primer	Forward primer
*MYD88*	AAACTGCGAGTGGGGTCAG	CATGTTCTCCATACCCTTGGT
*GAPDH*	TGGGTGGTCCAGGGTTTCTTACTCCTT	CGACTTCAACAGCAACTCCCACTCTTCC
*TBP*	CACATCACAGCTCCCCACCA	TGCACAGGAGCCAAGAGTGAA

### Statistical analysis

Whenever possible, results were expressed as mean ± standard error of the mean (SEM) or standard deviation (SD). The Statistical Package for Social Science (SPSS version 17.0) was used for data analysis. Categorical variables were compared with the chi-square test, and continuous variables with the Student’s t-test (age) or the Mann Whitney test (gene expression levels between two independent groups). Rivastigmine-treated and non-treated mice were compared regarding the effect of medication on *MYD88* transcript levels at T1 and T2 by Repeated Measures ANOVA with the Bonferroni Corrected t-tests.

## Results

**Fig. 1:**
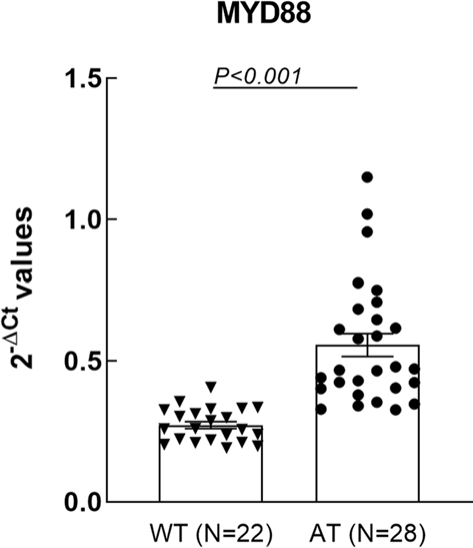
The mRNA levels of *MYD88*±SEM in the blood of AT (N = 28) and WT (N = 22) mice (FC = 2.04, p < 0.001). Comparison between mice groups was performed with the Mann Whitney test, and differences were considered significant for p < 0.05

**Fig. 2:**
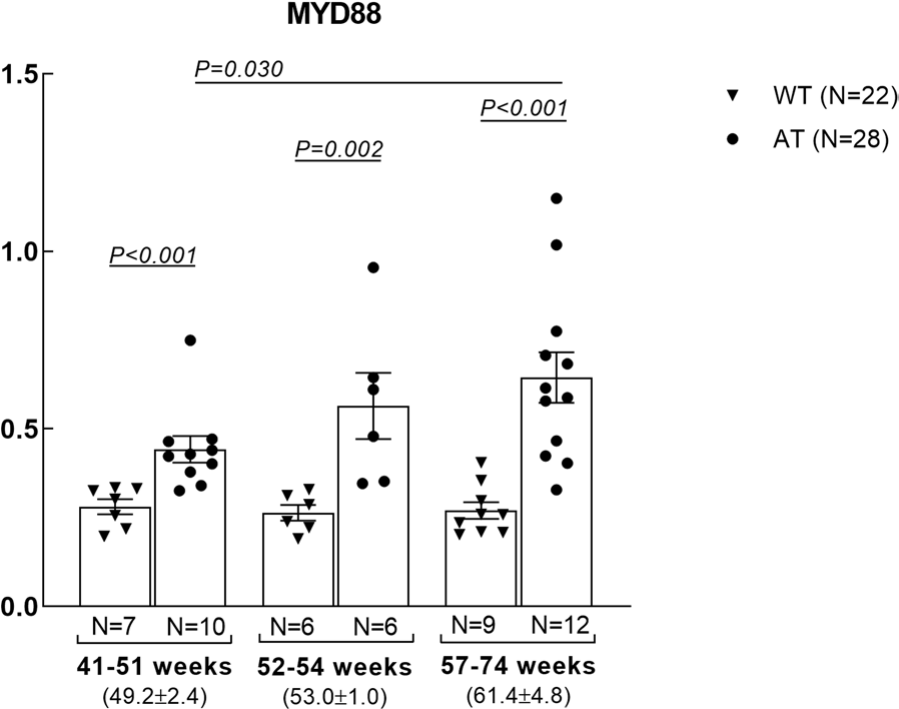
*MYD88* mRNA levels ±SEM in the blood of WT (N = 22) and AT (N = 28) mice in different age categories. Comparison between groups was performed using the Mann Whitney test, and differences were considered significant for p < 0.05

The expression analysis on *MYD88* in whole blood showed that the gene was significantly upregulated in AT mice as compared to WT controls (FC = 2.04, p < 0.001) (Fig. 1). The two groups were homogenous for age and sex. However, since AD transgenic female mice have been shown to be more susceptible to amyloid beta plaques and tangles and also TAU pathology compared to the males [21], an additional analysis has been performed. Comparing the MYD88 blood levels between the AT transgenic males (N = 18) vs. females (N = 10) no significant differences were observed (FC = 1.01, p = 1).To investigate a potential dependence of the registered gene over-expression on age, mice in each group were divided in three age categories, as follows: young-middle aged (41–51 weeks), middle aged (52–54 weeks) and old-middle aged (57–74 weeks) mice, and *MYD88* levels were analyzed accordingly. Results evidenced a significant *MYD88* upregulation in the AT group as compared to WT mice in each of the considered age categories (Fig. 2). Within the AT mice group, an increase of *MYD88* levels was observed especially in old-middle aged mice when compared to young-middle aged (p = 0.030) (Fig. 2). Since such an upregulation was not detected in the control WT group (Fig. 2), results suggested that *MYD88* overexpression was not age-dependent, but the levels of transcript are apparently increasing in time due to disease progression.

**Fig. 3:**
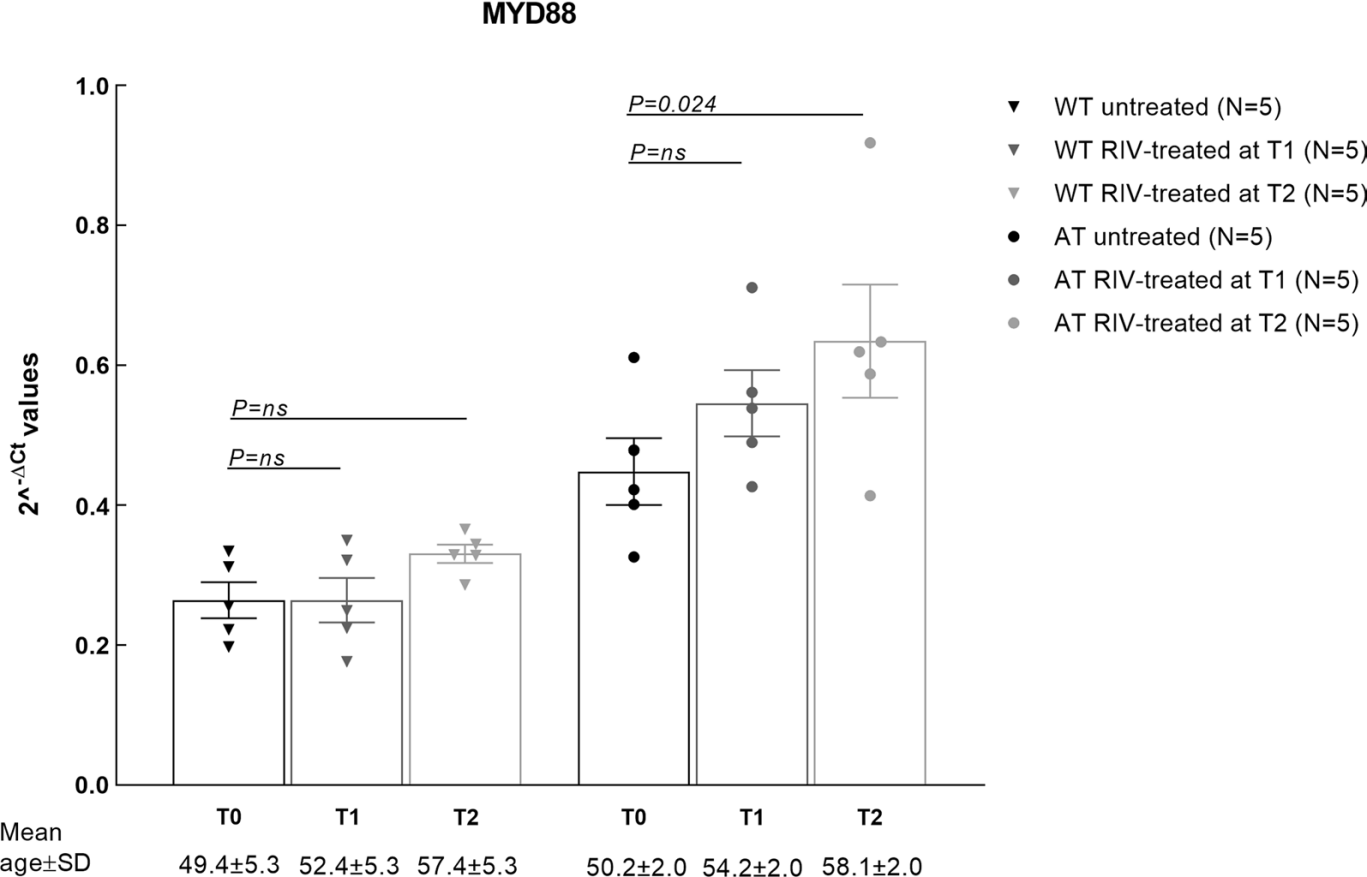
*MYD88* mRNA blood levels at different time points before and during Rivastigmine treatment: T0-before treatment, T1-at 20 days, T2-at 50 days after treatment initiation. The mean±SD of mice age at the investigated time points during therapy is mentioned. Bars represent the mean of *MYD88* levels±SEM. Comparison among T0, T1 and T2 was performed using Repeated Measures ANOVA with the Bonferroni Corrected t-tests, and differences were considered significant for p < 0.05. *RIV* Rivastigmine

Considering that AD patients are generally treated with acetylcholinesterase inhibitors, such as Rivastigmine, we investigated if Rivastigmine treatment could influence the transcript levels of *MYD88* in the examined mice groups. Five AT and five WT mice were treated by daily oral gavage with Rivastigmine (0.75 mg/kg) for 50 days, and blood *MYD88* mRNA levels were measured at baseline (T0), at 20 (T1) and 50 days (T2) of treatment. Results showed that Rivastigmine did not significantly change *MYD88* transcript levels in the WT group, after neither 20 nor 50 days of therapy (Fig. 3). An increase of *MYD88* mRNA levels was registered in the AT group only at 50 days of therapy (Fig. 3). This increase was most probably related to the disease evolution, and not to therapy (Fig. 3), considering the same ascending trend without (Fig. 2) or with Rivastigmine therapy.

## Discussion

*MYD88*, encoding for a key signal transductor in sterile inflammation, was found significantly upregulated in AD double transgenic mice, partially mimicking human AD, as compared to WT control mice. The increased of *MYD88* mRNA levels was not age-dependent, but apparently the transcript levels increased during disease progression. To our knowledge, this is the first study investigating blood mRNA levels of *MYD88* in an AD mice model. Moreover, we did not find so far human studies focused on blood *MYD88* levels in AD. The increased blood levels of *MYD88*, evidenced in the present study, indicate that blood leukocytes from AD transgenic mice, might have an increased susceptibility to respond to TLR ligands, either Pathogen-Associated Molecular Patterns (PAMPs) or Damage-Associated Molecular Patterns (DAMPs) [22, 23]. This could increase the immune competence to fight infection and to repair tissue damages, but may also perpetuate through a vicious cycle systemic sterile inflammation when the coordinated leukocyte effort cannot clear the immunostimulatory cues [24, 25]. The pathologic significance of the reported increase of *MYD88* transcript levels in blood was not investigated in the present study. Moreover, it is not clear, if the observed increase in gene expression is translated into higher protein levels of MYD88, or if such an overexpression has functional significance for immune homeostasis.

Preclinical and clinical evidence stated that MYD88 levels are increased in the AD brain. Immunofluorescence studies on 5XFAD mice showed higher MYD88 levels in the hippocampus and cortex of these transgenic mice, resulting in increased protein levels [13]. The authors found similar results also in humans, as MYD88 was increased in post mortem brains from AD patients as compared to MCI or to non-demented controls, and MYD88 protein levels positively correlated with the Braak staging. Another study, performed on the APP/PS1 mouse model, showed that neural stem cell transplantation decreased neuroinflammation and induced cognitive improvement, accompanied by a decreased expression of the MYD88 protein [26]. Moreover, the deletion of one *MYD88* allele in APP/PS1 transgenic mice was associated with a decrease of cerebral Aβ load and with an improvement of cognition [15]. Additionally, MYD88 levels were found increased also in the hippocampus of Balb/c mice where AD-like dementia was induced through the administration of Aβ1-42 oligomers [27]. In this study, MYD88 levels were down-regulated by minocycline treatment that ameliorated cognitive impairment, possibly through its anti-inflammatory action.

Considering the data evidencing increased levels of MYD88 in the AD brain, it is possible that neuronal damage in AD and the consequent release of DAMPs could have an echo in the periphery and prime blood leukocytes [28] by increasing the transcription of *MYD88*, as reported in the present study. Alternatively, circulating inflammatory factors or activated/primed inflammatory blood cells arising from a peripheral or distant inflammation might be recruited in the AD brain and increase locally the inflammatory microenvironment [29].

Although, the mechanisms underlying the brain-blood connection relative to MYD88 is not known, this adaptor protein might represent a valuable therapeutic target [24, 30–32], albeit being hypothetically beneficial in AD, raises concerns due to its critical role in anti-infectious defence. In this context, we have investigated if the AD-specific treatment with Rivastigmine, an acetylcholinesterase inhibitor, having also anti-inflammatory properties [33], is impacting on *MYD88* transcript levels in the blood of the investigated AT mice. Albeit its known anti-inflammatory action, Rivastigmine treatment could not decrease the mRNA levels of *MYD88* neither in AT nor in WT mice, probably because an unknown inflammatory stimulus, not affected by Rivastigmine, is decisively dictating gene over-expression.

## Conclusions

In conclusion, the study emphasized elevated transcript levels of the *MYD88* gene in the blood of double transgenic AT mice used as AD animal model. *MYD88* expression increased during disease evolution, and was not affected by Rivastigmine therapy. Accordingly, our results point towards *MYD88* as a promising blood biomarker to monitor AD progression, which has to be further validate in human studies.

## Supplementary Information

**Additional file 1.** All data generated or analyzed during this study are included in this published article and its additional information files. The mRNA data are also available in the Harvard Dataverse repository https://doi.org/10.7910/DVN/XRQWSE.

## Data Availability

All data generated or analyzed during this study are included in this published article and its additional information files. The mRNA data are also available in the Harvard Dataverse repository 10.7910/DVN/XRQWSE.

## Abbreviations

AD: Alzheimer’s disease
ALS: Amyotrophic lateral sclerosis
APP: β-Amyloid precursor protein
AT: APP/TAU
CNS: Central nervous system
DAMPs: Damage-associated molecular patterns
IRAK: Interleukin-1 receptor-associated kinase
MYD88: Myeloid differentiation factor-88 adaptor protein
NFB: Nuclear factor-kappa B
PAMPs: Pathogen-Associated Molecular Patterns
PD: Parkinson disease
TLR: Toll-like receptor
WT: Wild type
